# Strategies for the Prevention of Medication-Related Osteonecrosis of the Jaw (MRONJ): An Umbrella Review

**DOI:** 10.7759/cureus.95801

**Published:** 2025-10-31

**Authors:** Amina Gharibi, Zineb Essadeq, Hajar Yamine, Zineb Al Jalil, Jamila Kissa

**Affiliations:** 1 Periodontology, Faculty of Dental Medicine, Hassan II University of Casablanca, Casablanca, MAR; 2 General Dentistry, Faculty of Dental Medicine, Hassan II University of Casablanca, Casablanca, MAR; 3 Pediatric Dentistry, Faculty of Dental Medicine, Hassan II University of Casablanca, Casablanca, MAR

**Keywords:** antiresorptive drugs, jaws osteonecrosis, medication-related osteonecrosis of the jaw, oral surgery, prevention, risk factors

## Abstract

The objective of this review is to evaluate the effectiveness of different preventive strategies for medication-related osteonecrosis of the jaw (MRONJ) in at-risk patients. This umbrella review was carried out using six electronic databases: PubMed, Cochrane Library, Google Scholar, ScienceDirect, Mendeley, and Scopus. Moreover, it followed the Preferred Reporting Items for Systematic Reviews and Meta-Analyses (PRISMA) 2020 guidelines. The results of the clinical trials were described according to the PICO (Patient/Population/Problem, Intervention, Comparison, and Outcome) criteria. Prevention strategies for MRONJ discussed in the review include local preventive measures before starting antiresorptive treatment, as well as preventive measures during invasive procedures in patients undergoing antiresorptive treatment, such as temporary discontinuation of antiresorptive treatment, prophylactic antibiotic therapy, use of autologous platelet concentrates, laser therapy, surgical measures promoting healing, and, finally, the possibility of combinations of these preventive protocols. Our umbrella review does not allow us to determine the validity of the different preventive protocols for MRONJ in patients undergoing antiresorptive or anti-angiogenic treatment due to the lack of evidence in the systematic reviews included, the diversity of clinical situations, and the absence of direct comparison between the different strategies. Randomized clinical trials are needed to establish evidence-based recommendations.

## Introduction and background

Medication-related osteonecrosis of the jaw (MRONJ) is a relatively rare condition, characterized by exposed jawbone that fails to heal over a typical period in patients exposed to these medications, without prior jaw irradiation. MRONJ is associated with prolonged use of antiresorptive and antiangiogenic agents and, although uncommon, has a substantial impact on oral function and care pathways. It is primarily linked to antiresorptives (e.g., bisphosphonates, denosumab) and, to a lesser extent, anti-angiogenic agents. The risk varies between high-dose oncology regimens and low-dose osteoporosis therapy, highlighting the need for tailored prevention strategies.

Initially termed bisphosphonate-related osteonecrosis of the jaw (BRONJ), this complication was first described in 2003 by Marx [1]. In 2014, due to the increasing variety of implicated agents, the American Association of Oral and Maxillofacial Surgeons (AAOMS) adopted the broader nomenclature MRONJ [2]. 

Regarding antiresorptive agents, bisphosphonates such as alendronate, risedronate, ibandronate, and zoledronic acid act by inhibiting bone resorption and are commonly used to treat cancer-related conditions, including tumor-induced hypercalcemia, spinal cord compression, and bone fractures associated with skeletal metastases. They also play a critical role in the prevention of osteoporotic fractures [3].

Denosumab, another antiresorptive drug, is used to prevent fractures in patients with osteoporosis or skeletal metastases [3]. RANK ligand inhibitors, which function as antiangiogenics, are employed in the management of giant cell tumors and fibrous dysplasia [3]. Romosozumab, a novel monoclonal antibody, is used to prevent fractures in postmenopausal women with osteoporosis by stimulating bone formation [3]. Recent studies have also implicated other drug classes as potential MRONJ risk factors [4-7]. These include tyrosine kinase inhibitors (e.g., sunitinib), monoclonal antibodies (e.g., bevacizumab), fusion proteins (e.g., aflibercept), mTOR inhibitors (e.g., everolimus), radiopharmaceuticals (e.g., radium-223), selective estrogen receptor modulators (e.g., raloxifene), and immunosuppressants (e.g., methotrexate and corticosteroids). MRONJ must be differentiated from other forms of osteonecrosis.

Although antiresorptive and antiangiogenic drugs offer substantial therapeutic benefits, they are associated with a potentially severe adverse effect. Therefore, it is imperative to develop effective preventive strategies that allow the continued use of these medications while minimizing the risk of complications. It should be emphasized that not all patients receiving these treatments develop MRONJ. Other factors can also play a role in the development of MRONJ, such as the use of concomitant medications (e.g., tyrosine kinase inhibitors), systemic conditions like diabetes, and local oral factors, including poor oral hygiene or dental extractions. The condition may occur spontaneously or be triggered by invasive dental procedures [8]. 

The etiology and pathogenesis of MRONJ remain incompletely understood, though several risk factors have been identified. These include the type of drug administered, cumulative drug dose, and poor oral hygiene [2]. Some studies have concluded that tooth extraction is the most significant independent risk factor for MRONJ onset, as the procedure induces direct trauma to the alveolar bone and disrupts mucosal integrity, facilitating microbial contamination [2,9]. In patients treated with antiresorptive or antiangiogenic medications, these effects are exacerbated by impaired bone remodeling and angiogenesis, leading to delayed healing and increased risk of osteonecrosis. Additionally, local infection and inflammation at the extraction site can exacerbate tissue damage. Consequently, invasive procedures such as tooth extractions should be avoided whenever possible in patients receiving high doses of antiresorptive agents [2].

Prevention is the key stone in reducing MRONJ incidence. In the clinical management of at-risk patients, a preventive approach should always be prioritized [10]. For cancer patients scheduled to receive bone-modifying agents (BMAs) or antiangiogenic drugs in a non-urgent setting, a comprehensive oral examination, including dental, periodontal, and radiographic assessments, should be performed prior to initiating therapy [2].

Indeed, patients who receive adequate dental care and maintain optimal oral health over time exhibit a lower risk of developing MRONJ [2,11]. A coordinated dental care plan should be established between the dentist and the oncologist to ensure that all medically necessary dental procedures are completed before the initiation of BMAs [12].

The aim of our study is to investigate the various preventive strategies for MRONJ through an umbrella review. This type of study synthesizes findings from systematic reviews and meta-analyses addressing the same research objective. It allows for a comprehensive overview of existing preventive protocols and the identification of emerging strategies.

The research question guiding our umbrella review is as follows: What is the validity of the different preventive protocols for MRONJ?

## Review

Methods

This study is an umbrella review conducted in accordance with the Preferred Reporting Items for Systematic Reviews and Meta-Analyses (PRISMA) 2020 guidelines [13]. The methodology was developed based on a systematic review framework to ensure rigor and transparency. A comprehensive literature search was carried out using six major databases: PubMed, Cochrane Library, Google Scholar, ScienceDirect, Mendeley, and Scopus.

The search strategy was structured around three main keyword blocks. Block 1 focused on terms related to the condition of interest, including "jaw necrosis", "necrosis", "osteonecrosis", "bone lesions", and "bone complications". Block 2 targeted preventive aspects, with keywords such as "prophylaxis", "prevention", "preventive treatment", "preventive measures", and "preventive interventions". Block 3 addressed the therapeutic agents involved, including "bisphosphonates", "denosumab", "anti-RANKL", "corticosteroids", "drugs", "tyrosine kinase inhibitors", and "antineoplastic agents". In addition, a Medical Subject Headings (MeSH)-based search equation was applied to ensure the inclusion of relevant indexed literature. The equation used was as follows: (((("Jaw"[Mesh]) AND "Necrosis"[Mesh]) OR ("Osteonecrosis"[Mesh])) AND (("prevention and control" [Subheading]) OR "Primary Prevention"[Mesh])) AND ((((((("Diphosphonates"[Mesh]) OR "Denosumab"[Mesh])) OR "Adrenal Cortex Hormones"[Mesh]) OR "Pharmaceutical Preparations"[Mesh]) OR "Tyrosine Kinase Inhibitors"[Mesh]) OR "Antineoplastic Agents"[Mesh]).

To refine the results, filters were applied to include only meta-analyses and systematic reviews published between 2005 and 2023. The inclusion criteria were as follows: systematic reviews or meta-analyses focusing on interventions, preventive measures, or protocols specifically related to the primary prevention of osteonecrosis of the jaw (ONJ); studies including adult populations; and publications written in English or French within the defined timeframe. Exclusion criteria included articles for which full texts could not be obtained, even after contacting authors via email, as well as systematic reviews based on animal studies or in vitro experiments.

Results

The electronic search yielded a total of 2901 titles across all six databases. Specifically, the PubMed search using Boolean operators identified 504 articles. The Cochrane Library search returned 10 articles. Google Scholar produced the highest number of results, with 2160 articles after applying the relevant filters. The search on ScienceDirect retrieved 123 articles, while Mendeley and Scopus provided 24 and 80 articles, respectively.

**Figure 1:**
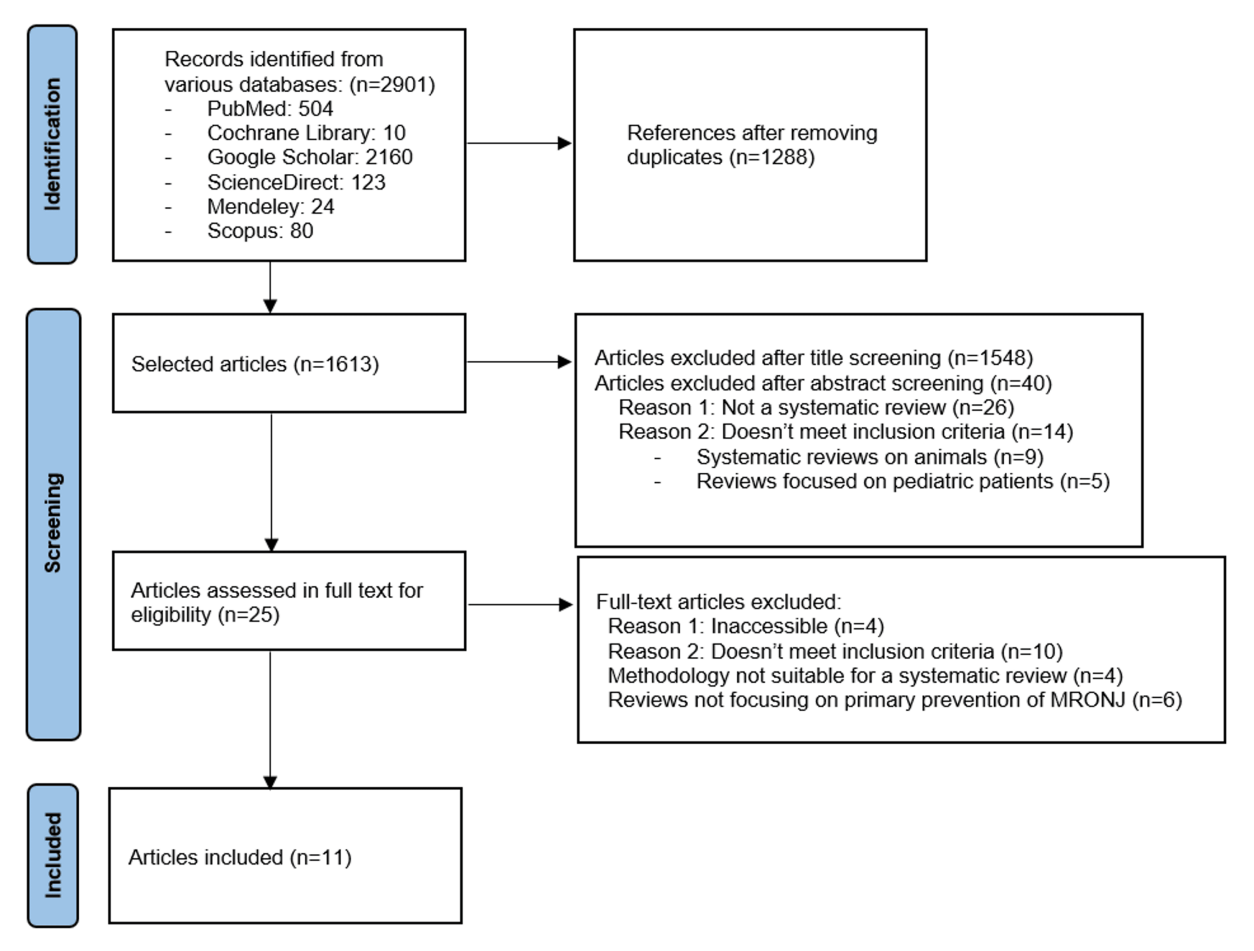
PRISMA flow diagram PRISMA: Preferred Reporting Items for Systematic Reviews and Meta-Analyses; MRONJ: medication-related osteonecrosis of the jaw

After screening the titles of the 1613 articles that made up the initial raw dataset, 65 articles were selected for full-text evaluation. A total of 1548 articles were excluded based on the predefined exclusion criteria. The article selection process is illustrated in Figure 1. Following the full-text review, 11 systematic reviews met all eligibility criteria and were included in this umbrella review. Each of these reviews covered a specific time period and focused on the preventive management of ONJ. Summary information and characteristics of the included reviews are presented below.

Characteristics of the Included Systematic Reviews

A total of 11 systematic reviews were included in this umbrella review (Tables 1-2). All systematic reviews covered a defined time period. Regarding language restrictions, five reviews did not report this characteristic [14-18], four reviews applied a language restriction [19-22], and two reviews had no language restrictions [23,24]. Some systematic reviews also addressed the treatment of MRONJ in conjunction with prevention strategies [15,17,20,22-24].

**Table 1:** PICO characteristics of the included systematic reviews PICO: Population, Intervention, Comparison, and Outcome; BP: bisphosphonate; OB: oral bisphosphonate; IB: intravenous bisphosphonate; AR: antiresorptive; MRONJ: medication-related osteonecrosis of the jaw; PRGF: platelet-rich growth factor; PRF: platelet-rich fibrin; CPA: platelet aggregates; BRONJ: bisphosphonate-related osteonecrosis of the jaw; ONJ: osteonecrosis of the jaw; PRP: platelet-rich plasma; aPDT: antimicrobial photodynamic therapy; L-PRF: leukocyte-rich platelet fibrin; PBMT: photobiomodulation therapy

Authors (year)	Population	Intervention (exposure)	Comparison	Outcome	Conclusion
Del Fabbro et al. (2015) [23]	4 studies: 219 patients treated with IB and 1 with OB	Tooth extractions with or without platelet concentrates at the extraction site	In one study: test group (extraction + platelet concentrates) vs. control (extraction only); three studies without control	CPA use may lower BRONJ risk (97.26% success). No difference by jaw/gender	Autologous platelet concentrates (CPA) may reduce BRONJ risk in BP-treated patients, though evidence is limited
Diniz-Freitas and Limeres (2016) [14]	13 studies: 634 IB patients and 1261 OB patients	Preventive protocols: antibiotics, atraumatic extractions, PRGF, laser therapy, and chlorhexidine rinses	No control group	MRONJ: 6.9% (IV) vs. 0.47% (oral). Preventive measures reduced risk; no ONJ in 700 oral BP patients	No definitive evidence supports current prevention protocols after extractions in AR patients
Lopez-Jornet et al. (2016) [15]	3 studies: 218 IB patients	Tooth extractions with PRGF	No control group	7 ONJ cases after 697 extractions (5 mandible, 2 maxilla)	Evidence on platelet concentrates is limited
Poxleitner et al. (2017) [19]	12 studies: 4457 cancer patients (IB or OB)	Oral hygiene before BP therapy, prophylactic antibiotics, flap closure, and laser/PRGF	No control group	Dental care reduced ONJ from 4.6% to 0.8%; antibiotics lowered ONJ risk; MRONJ: 2.5% (laser) and 2.3% (PRGF)	Pre-treatment oral hygiene and conservative surgery reduce MRONJ incidence
Karna et al. (2018) [16]	6 studies: 2332 cancer patients (IB or OB)	Before AR therapy: dental check and oral hygiene. During therapy: antibiotics (7 days) and PRGF for extraction	4 retrospective and 2 prospective without preventive treatments	Preventive measures reduced MRONJ by 77.3%	Preventive dental interventions significantly lower MRONJ in cancer patients
Fortunato et al. (2020) [20]	8 studies: 262 with CPA and 185 controls; 237 implant patients with CPA	Platelet concentrates (PRP, PRGF, PRF) for extraction/implant placement ± antibiotics	Two studies had control group without CPA	MRONJ: 0.9% CPA vs. 1.2% control; 0% after implant placement	CPA effectiveness uncertain; may help via immunomodulatory effect
Ottesen et al. (2020) [17]	14 studies: 2100 high-dose AR patients	Drug holiday during extraction/surgery	Continuation of AR treatment	No difference in MRONJ risk between continuation and pause. Retrospective studies mixed	No strong evidence supporting drug holiday; case-by-case advised
Romero-Ruiz et al. (2021) [18]	Patients on AR (OB or IB)	Prevention protocol, antibiotic coverage, infection control, and extractions under coverage	-	Preventive protocols reduced MRONJ: 22.3%→2.2% and 3.2%→1.3%	Zoledronate increases MRONJ risk; prevention and education reduce it
Cabras et al. (2021) [21]	17 studies: 462 IB, 1227 OB, and 19 denosumab patients	Antibiotics, atraumatic surgery, tissue closure, antiseptic rinses, and laser	No treatment/placebo/other antibiotics	IB with closure: 0-13.1%; without: 5-35%. OB with closure: 0.1%; without: 1%. Denosumab: 2.5%	Antibiotics may reduce MRONJ; closure not significantly different
Beth-Tasdogan et al. (2017) [24]	1366 IB/OB participants	(1) Regular dental exams and preventive care. (2) PRGF during extraction. (3) Surgical flap closure. (4) Chlorhexidine + antibiotics. (5) Subperiosteal vs. epiperiosteal closure	Standard care/no PRGF/other surgical methods	Preventive care ↓ MRONJ 90%. PRGF group: 0 MRONJ vs. 5 in control. Flap techniques: no MRONJ	PRGF and preventive protocols may reduce MRONJ; further evidence needed
Scribante et al. (2023) [22]	BP-treated patients (oral or IV)	Autologous platelet concentrates ± laser therapy	With or without a comparison group	Laser + autologous platelet concentrates prevented MRONJ after 6 months; aPDT safe; L-PRF + PBMT improved healing	Autologous platelet concentrates and laser enhance healing and reduce MRONJ risk, promising combined approach

**Table 2:** Bibliometric characteristics of the included systematic reviews SR: systematic review; MA: meta-analysis; MRONJ: medication-related osteonecrosis of the jaw; BRONJ: bisphosphonate-related osteonecrosis of the jaw; APC: autologous platelet concentrate; PRGF: plasma-rich growth factors; PRP: platelet-rich plasma; PRF: platelet-rich fibrin; AR: antiresorptive agent; RCT: randomized controlled trial

Authors (year)	Type and number of included studies	Qualitative assessment tool	Analysis method	Studied variables/main findings
Del Fabbro et al. (2015) [23]	4 prospective studies; 2 case series; 1 case-control study; 1 case report	Not mentioned	SR and MA	Incidence of BRONJ after oral surgery; use of APC in the prevention of BRONJ after extraction
Diniz-Freitas and Limeres (2016) [14]	13 observational studies (8 prospective, 5 retrospective)	Not mentioned	SR	Evaluation of preventive protocols for MRONJ; incidence and effectiveness of preventive measures
Lopez-Jornet et al. (2016) [15]	3 clinical trials	Newcastle-Ottawa Scale	SR	Use of APC for MRONJ prevention; incidence of MRONJ
Poxleitner et al. (2017) [19]	9 prospective studies; 3 retrospective studies	Cochrane Tool	SR	Incidence of MRONJ; effectiveness of preventive measures such as oral hygiene, antibiotics, and modified surgical techniques
Karna et al. (2018) [16]	6 clinical studies (1 case-control, 1 RCT, 4 cohort)	Cochrane Tool	SR and MA	Effectiveness of preventive dental interventions before treatment (oral exam, dental care, hygiene instructions) and pre-extraction measures (antibiotics, PRGF); MRONJ incidence
Fortunato et al. (2020) [20]	8 studies (2 case reports, 1 case series, 1 retrospective, 2 prospective, 2 clinical trials)	Jadad Scale	SR	Incidence of MRONJ; effectiveness of APC in MRONJ prevention after extraction
Ottesen et al. (2020) [17]	14 observational studies (3 prospective, 11 retrospective)	Newcastle-Ottawa Scale	SR	Effectiveness of the temporary discontinuation ("drug holiday") of AR before surgery to prevent MRONJ in cancer patients
Romero-Ruiz et al. (2021) [18]	21 articles (10 clinical, 11 reviews)	Not mentioned	SR	Comparison of preventive protocols in patients exposed to AR or antiangiogenic drugs; MRONJ prevention strategies
Cabras et al. (2021) [21]	17 studies (9 prospective, 1 cohort, 4 retrospective, 3 case series >10 cases)	QUIPS Tool	SR	Effect of systemic antibiotics on MRONJ risk; incidence after extractions; influence of extraction techniques (closure, antimicrobials, photobiomodulation)
Beth-Tasdogan et al. (2017) [24]	5 randomized clinical trials	Cochrane Tool	SR and MA	Type of interventions for MRONJ prevention; incidence of MRONJ
Scribante et al. (2023) [22]	6 randomized clinical trials	Cochrane Tool	SR	Use of APC (PRP, PRF) and/or laser therapy for MRONJ prevention

Qualitative Assessment of Risk of Bias in the Included Systematic Reviews

Only one of the included systematic reviews fully met the AMSTAR 2 criteria [24]. In total, two reviews were rated as low quality [16-20], while eight were assessed as having critically low quality [14,15,17-19,21-23] (Table 3).

**Table 3:** Qualitative assessment of the risk of bias in the included systematic reviews based on the AMSTAR 2 criteria PY: partially yes; -: no meta-analysis was conducted

Authors (year)	1	2	3	4	5	6	7	8	9	10	11	12	13	14	15	16	Quality of review
Del Fabbro et al. (2015) [23]	No	No	No	PY	Yes	Yes	PY	PY	No	No	No	No	No	Yes	No	Yes	Very low
Diniz-Freitas and Limeres (2016) [14]	Yes	No	No	PY	Yes	No	No	No	No	No	-	-	No	No	-	Yes	Very low
Lopez-Jornet et al. (2016) [15]	No	PY	No	PY	Yes	Yes	No	No	PY	No	-	-	No	Yes	-	Yes	Very low
Poxleitner et al. (2017) [19]	No	PY	No	PY	Yes	No	No	No	Yes	No	-	-	No	No	-	Yes	Very low
Karna et al. (2018) [16]	Yes	Yes	No	PY	Yes	Yes	No	PY	Yes	Yes	Yes	Yes	Yes	Yes	Yes	Yes	Low
Fortunato et al. (2020) [20]	Yes	PY	No	PY	Yes	Yes	No	PY	Yes	No	-	-	No	No	-	Yes	Low
Ottesen et al. (2020) [17]	Yes	PY	No	PY	Yes	No	No	PY	No	Yes	-	-	Yes	No	-	Yes	Very low
Romero-Ruiz et al. (2021) [18]	No	No	No	PY	Yes	No	No	No	No	No	-	-	No	No	-	Yes	Very low
Cabras et al. (2021) [21]	Yes	PY	No	PY	Yes	Yes	No	PY	Yes	No	-	-	No	Yes	-	Yes	Very low
Beth-Tasdogan et al. (2017) [24]	Yes	Yes	No	PY	Yes	Yes	PY	Yes	Yes	Yes	Yes	Yes	Yes	Yes	Yes	Yes	Low
Scribante et al. (2023) [22]	Yes	PY	No	PY	No	No	No	No	PY	Yes	-	-	No	No	-	Yes	Very low

Discussion

Several systematic reviews have highlighted that those local preventive measures, such as oral cavity disinfection, should be implemented before starting treatment with antiresorptive or antiangiogenic medications. A thorough examination of the oral cavity by a dentist is essential before initiating antiresorptive treatment, as emphasized in the systematic review by Poxleitner et al. [19]. It is crucial for patients to be informed about the risk of MRONJ, and regular follow-ups every six months are recommended. It's also recommended to remove non-restorable teeth and doomed implants and adjust dental prostheses to prevent mucosal injuries [19].

Karna et al. [16] have also concluded that these measures may reduce the need for future extractions, thus minimizing the risk of MRONJ [16]. An interdisciplinary approach is required for the implementation of these protocols, involving a qualified team of dentists, hygienists, and surgeons, as well as collaboration with other medical professionals managing the underlying condition [16].

A randomized trial in the meta-analysis by Beth-Tasdogan et al. [24] compares regular dental examinations (every three months) and preventive treatments against standard care in patients who have already started antiresorptive treatment. The results indicate a significant reduction in the incidence of MRONJ with preventive treatments.

The consensus document published by the American Association of Oral and Maxillofacial Surgeons in 2009 recommended interrupting treatment three months before and three months after tooth extraction, but only for patients who had received oral bisphosphonates for more than four years, provided the patient's systemic conditions allowed for it [2]. The 2014 update of this document reduced the therapeutic interruption period before extraction to two months.

The systematic reviews conducted by Diniz-Freitas and Limeres [14], Karna et al. [16], and Ottesen et al. [17] extensively explore the effect of drug holiday in the prevention of MRONJ. In the review by Diniz-Freitas and Limeres, only one study compared two groups of patients with or without drug interruption. The results showed one case of ONJ in the group without interruption, while no cases were reported in the group with a temporary cessation of three months before dental extraction [14]; however, this result was not statistically significant. This was also confirmed by Karna et al. in 2018, highlighting that there is little data to support or refute the benefits of a drug holiday [16].

The systematic review by Ottesen et al. in 2020 sheds light on the diversity of results observed in included studies, with some results favoring drug holiday but significant methodological limitations. The varying pharmacokinetic properties of antiresorptive agents, such as bisphosphonates and denosumab, underscore the importance of tailoring drug holiday decisions and protocols based on the specific medication [17].

The systematic review by Romero-Ruiz et al. [18] highlighted that antibiotic prophylaxis is widely accepted for any oral surgery intervention in patients treated with antiresorptive agents. This underscores the importance of antibiotic prophylaxis in preventing MRONJ.

Several systematic reviews, including those by Del Fabbro et al. [23] and Poxleitner et al. [19], have emphasized the importance of amoxicillin. This antibiotic is effective against *Actinomyces*, a microorganism often present in MRONJ lesions. The systematic review by Cabras et al. [21] noted that the most commonly used antibiotic dosage in the included studies is 2-3 g of amoxicillin per day, either alone or in combination with a beta-lactamase inhibitor or metronidazole. However, there is no uniform approach regarding the duration of antibiotic treatment before and after dental extraction, which varies from three to 20 days, with an average duration of seven days. The most common route of administration is oral. In some cases, alternative treatments are suggested, such as clindamycin or erythromycin, in cases of allergy to beta-lactam antibiotics. The prognosis can be favorable but remains variable and depends on individual patient factors [21].

Studies by Del Fabbro et al. [23], Diniz-Freitas and Limeres [14], Scribante et al. [22], Lopez-Jornet et al. [15], and Karna et al. [16] reported positive results regarding the use of autologous platelet concentrates (APCs), whether in the form of platelet-rich growth factor (PRGF), autologous plasma (AP), or leukocyte-rich platelet fibrin (L-PRF), following dental extractions in patients undergoing antiresorptive treatment. The authors noted a reduced incidence of MRONJ, or even none, in the groups treated with APCs compared to the control or non-APC groups.

Regarding implant surgery, Fortunato et al. [20] suggested that the use of APCs in this context may not present significant risks of MRONJ in patients on low-dose antiresorptive treatment. They observed no incidence of MRONJ over a long-term follow-up period. While these results are informative, they require cautious interpretation due to study limitations, such as variability in APC use protocols and potential biases.

It is noteworthy that antimicrobial photodynamic therapy (aPDT), unlike antibiotics, avoids side effects and is not associated with the development of bacterial resistance [22]. Systematic reviews by Diniz-Freitas and Limeres [14], Poxleitner et al. [19], and Scribante et al. [22] converge on the beneficial effects of laser therapy in wound healing and in reducing the risk of ONJ in patients receiving medications that affect bone metabolism. On the one hand, Scribante et al. [22] highlighted the positive effects of laser therapy on cellular proliferation and differentiation, as well as on wound healing. In patients with a history of bisphosphonate treatment, no incidence of MRONJ was reported six months after postoperative laser application. Furthermore, approaches such as aPDT and low-level laser therapy (LLLT) or photobiomodulation therapy (PBMT) are considered valuable adjuncts for enhancing post-extraction healing. Notably, aPDT, unlike antibiotics, avoids adverse effects and is not associated with the development of bacterial resistance. On the other hand, according to Diniz-Freitas and Limeres [14], the use of Nd:YAG laser therapy in combination with antibiotic prophylaxis significantly reduced the rate of ONJ in cancer patients receiving bisphosphonates, whether administered intravenously or orally. The review by Poxleitner et al. [19] also emphasized that the use of LLLT during and after tooth extraction led to a reduction in MRONJ incidence by 2.5%.

Furthermore, according to the findings of Diniz-Freitas and Limeres, the use of Nd:YAG laser in conjunction with antibiotic prophylaxis significantly reduced the rate of ONJ in cancer patients receiving bisphosphonates, whether administered intravenously or orally [14].

The review by Poxleitner et al. also highlights that the use of LLLT during and after extraction resulted in a reduction of MRONJ cases by 2.5% [19].

Systematic reviews conducted by Diniz-Freitas and Limeres [14] and Poxleitner et al. [19] found favorable results regarding protocols that combine antibiotic prophylaxis, atraumatic dental extractions, and the local application of antiseptics to reduce the risk of postoperative infection. Moreover, the systematic review by Poxleitner et al. [19] associated antibiotic prophylaxis with the application of PRGF in the extraction socket to promote healing, in addition to prophylactic antibiotic therapy. This intervention could be particularly beneficial for patients with compromised healing. The review by Cabras et al. also noted that after an alveolectomy, the antibiotic therapy and the placement of a biological membrane enriched with growth factors can significantly reduce the risk of MRONJ in patients on intravenous bisphosphonate, decreasing from 4.45% to 0.91% [21].

Furthermore, a study included in the review by Beth-Tasdogan et al. [24] examined the effects of a platelet-rich fibrin clot inserted into the extraction socket without primary closure compared to primary closure of the extraction socket with a mucosal flap after dental extraction. At the final control, in the examination conducted 90 days after the intervention, all participants showed complete mucosal closure without signs of MRONJ, demonstrating that this combination of protocols yielded positive results against MRONJ.

## Conclusions

Our umbrella review does not allow us to determine the best preventive strategy for MRONJ in patients on treatments that induce this condition due to the lack of high-quality evidence, the diversity of clinical situations, and the absence of direct comparisons among the different strategies. We therefore recommend that dentists consider the individual characteristics of each patient, the type and duration of medication treatment, the type and urgency of the surgical procedure, and the patient's preferences in selecting the most appropriate preventive strategy. We also recommend conducting large-scale randomized clinical trials with long-term follow-up to compare the various preventive strategies in patients undergoing antiresorptive treatments, using standardized and validated outcome criteria. 

## References

[1] Marx RE (2003). Pamidronate (Aredia) and zoledronate (Zometa) induced avascular necrosis of the jaws: a growing epidemic. J Oral Maxillofac Surg.

[2] Ruggiero SL, Dodson TB, Fantasia J, Goodday R, Aghaloo T, Mehrotra B, O'Ryan F (2014). American Association of Oral and Maxillofacial Surgeons position paper on medication-related osteonecrosis of the jaw-2014 update. J Oral Maxillofac Surg.

[3] Ruggiero SL, Dodson TB, Aghaloo T, Carlson ER, Ward BB, Kademani D (2022). American Association of Oral and Maxillofacial Surgeons' position paper on medication-related osteonecrosis of the jaws-2022 update. J Oral Maxillofac Surg.

[4] Fusco V, Santini D, Armento G, Tonini G, Campisi G (2016). Osteonecrosis of jaw beyond antiresorptive (bone-targeted) agents: new horizons in oncology. Expert Opin Drug Saf.

[5] Nicolatou-Galitis O, Kouri M, Papadopoulou E (2019). Osteonecrosis of the jaw related to non-antiresorptive medications: a systematic review. Support Care Cancer.

[6] King R, Tanna N, Patel V (2019). Medication-related osteonecrosis of the jaw unrelated to bisphosphonates and denosumab-a review. Oral Surg Oral Med Oral Pathol Oral Radiol.

[7] Sacco R, Shah S, Leeson R, Moraschini V, de Almeida Barros Mourão CF, Akintola O, Lalli A (2020). Osteonecrosis and osteomyelitis of the jaw associated with tumour necrosis factor-alpha (TNF-α) inhibitors: a systematic review. Br J Oral Maxillofac Surg.

[8] Feng Z, An J, Zhang Y (2021). Factors influencing severity of medication-related osteonecrosis of the jaw: a retrospective study. J Oral Maxillofac Surg.

[9] Hallmer F, Andersson G, Götrick B, Warfvinge G, Anderud J, Bjørnland T (2018). Prevalence, initiating factor, and treatment outcome of medication-related osteonecrosis of the jaw-a 4-year prospective study. Oral Surg Oral Med Oral Pathol Oral Radiol.

[10] Dimopoulos MA, Kastritis E, Bamia C (2009). Reduction of osteonecrosis of the jaw (ONJ) after implementation of preventive measures in patients with multiple myeloma treated with zoledronic acid. Ann Oncol.

[11] Di Fede O, Panzarella V, Mauceri R (2018). The dental management of patients at risk of medication-related osteonecrosis of the jaw: new paradigm of primary prevention. Biomed Res Int.

[12] Yuan P (2021). Diagnosis and treatment of medication-related osteonecrosis of the jaws from an oncologist's perspectives [Article in Chinese]. Zhonghua Kou Qiang Yi Xue Za Zhi.

[13] Page MJ, McKenzie JE, Bossuyt PM (2021). The PRISMA 2020 statement: an updated guideline for reporting systematic reviews. BMJ.

[14] Diniz-Freitas M, Limeres J (2016). Prevention of medication-related osteonecrosis of the jaws secondary to tooth extractions. A systematic review. Med Oral Patol Oral Cir Bucal.

[15] Lopez-Jornet P, Sanchez Perez A, Amaral Mendes R, Tobias A (2016). Medication-related osteonecrosis of the jaw: is autologous platelet concentrate application effective for prevention and treatment? A systematic review. J Craniomaxillofac Surg.

[16] Karna H, Gonzalez J, Radia HS, Sedghizadeh PP, Enciso R (2018). Risk-reductive dental strategies for medication related osteonecrosis of the jaw among cancer patients: a systematic review with meta-analyses. Oral Oncol.

[17] Ottesen C, Schiodt M, Gotfredsen K (2020). Efficacy of a high-dose antiresorptive drug holiday to reduce the risk of medication-related osteonecrosis of the jaw (MRONJ): a systematic review. Heliyon.

[18] Romero-Ruiz MM, Romero-Serrano M, Serrano-González A, Serrera-Figallo MÁ, Gutiérrez-Pérez JL, Torres-Lagares D (2021). Proposal for a preventive protocol for medication-related osteonecrosis of the jaw. Med Oral Patol Oral Cir Bucal.

[19] Poxleitner P, Engelhardt M, Schmelzeisen R, Voss P (2017). The prevention of medication-related osteonecrosis of the jaw. Dtsch Arztebl Int.

[20] Fortunato L, Bennardo F, Buffone C, Giudice A (2020). Is the application of platelet concentrates effective in the prevention and treatment of medication-related osteonecrosis of the jaw? A systematic review. J Craniomaxillofac Surg.

[21] Cabras M, Gambino A, Broccoletti R, Sciascia S, Arduino PG (2021). Lack of evidence in reducing risk of MRONJ after teeth extractions with systemic antibiotics. J Oral Sci.

[22] Scribante A, Ghizzoni M, Pellegrini M, Pulicari F, Spadari F (2023). Laser devices and autologous platelet concentrates in prevention and treatment of medication-related osteonecrosis of the jaws: a systematic review. Medicina (Kaunas).

[23] Del Fabbro M, Gallesio G, Mozzati M (2015). Autologous platelet concentrates for bisphosphonate-related osteonecrosis of the jaw treatment and prevention. A systematic review of the literature. Eur J Cancer.

[24] Beth-Tasdogan NH, Mayer B, Hussein H, Zolk O (2017). Interventions for managing medication-related osteonecrosis of the jaw. Cochrane Database Syst Rev.

